# Strategic actions to advance multipurpose prevention technologies in low- and middle-income countries

**DOI:** 10.3389/frph.2023.1150857

**Published:** 2023-07-03

**Authors:** Bethany Young Holt, Ariane van der Straten, Taryn Barker, Z. Mike Chirenje, Anne-Isabelle Cameron, Cherise Scott, Carmen Pérez Casas, Joseph Romano

**Affiliations:** ^1^ CAMI Health, Initiative for MPTs, Public Health Institute, Sacramento, CA, United States; ^2^ Center for AIDS Prevention Studies (CAPS), Department of Medicine, UCSF, San Francisco, CA, United States and ASTRA Consulting, Kensington, CA, United States; ^3^ Children’s Investment Fund Foundation, London, United Kingdom; ^4^ Department of Obstetrics and Gynecology, UCSF, San Francisco, CA, United States; ^5^ UNITAID, Geneva, Switzerland; ^6^ NWJ Group, LLC, Wayne, PA, United States

**Keywords:** multipurpose prevention technologies, MPTs, HIV, LMICs (low and middle income countries), contraception, STIs

## Abstract

**Background:**

HIV, other sexually transmitted infections (STIs) and unintended pregnancies are critical and interlinked health risks for millions of women of reproductive age worldwide. Multipurpose prevention technologies (MPTs) offer an innovative approach for expanding combined pregnancy and/or disease prevention. So far, MPT development efforts have focused mostly on HIV prevention, but about half of product candidates comprise compounds active against non-HIV STIs as well. This review aims to provide a framework that promotes the efficient advancement of the most promising preclinical products through the development pathway and into the hands of end-users, with a focus on women in low- and middle-income countries (L/MICs).

**Methods:**

This mini review provides a summary of the current landscape of the MPT field. It comprises a landscape assessment of MPTs in development, complemented by a series of 28 in-depth, semi-structured key informant interviews (KIIs) with experts representing different L/MIC perspectives.

**Main results:**

We identified six primary action strategies to advance MPTs for L/MICs, including identification of key research gaps and priorities. For each action strategy, progress to date and key recommendations are included.

**Conclusions:**

To realize the life-saving potential of MPTs and maximize the momentum made to date, a strategic, collaborative and well-funded response to the gaps and next steps outlined in this paper is critical. A coordinated response can add rigor and efficiency to the development process, to successfully advance the most promising MPT products to the hands of end-users.

## 1. Introduction

For millions of women of reproductive age worldwide, HIV, other sexually transmitted infections (STIs) and unintended pregnancies are critical and interlinked health risks. Adolescent girls and young women (AGYW) from sub-Saharan Africa (SSA), aged 15–24, are at particular risk of HIV, representing 63% of all new HIV infections in 2021 (1). STIs are on the rise globally, leading to severe health consequences for women and their children, including pelvic inflammatory disease, infertility, ectopic pregnancy, chronic pelvic pain, and neonatal and infant infections (2). According to the World Health Organization (WHO), more than 1 million STIs are acquired every day globally (2, 3). Concurrently, an estimated 830 women die from preventable causes related to pregnancy and childbirth each day worldwide (4). More than 160 million women have an unmet need for contraception (5). Simultaneously, HIV stigma and other socio-structural barriers often discourage women from accessing biomedical HIV prevention strategies, such as pre-exposure prophylaxis (PrEP) (6).

Multipurpose prevention technologies (MPTs) are designed to deliver multifaceted prevention to address two or more of these health risks with a single product (7). Condoms are the only commercially available MPT; indeed, the majority of MPTs are in early stages of development. The MPT pipeline has grown over the past decade, primarily focused on combining anti-HIV drugs with hormonal contraceptive drugs into a single product (7). Given finite funding and technical challenges for the MPT field, the objective of this review is to provide a framework that promotes the most promising preclinical products through the development pathway and into the hands of end-users, with a focus on women in low- and middle-income countries (L/MICs).

## 2. Methodology

This review provides a summary of the previously published 60+ page landscape of MPT product candidates in all stages of preclinical and clinical development (6)*.* The search strategy included three principal avenues: product developer surveys, key informant interviews, and a desk review. Data collection was implemented between May and September 2021, with an update to the desk review in November–December 2022. The product developer surveys consisted of 18 questions about each MPT candidate included as part of the Initiative for MPT's (IMPT) annual MPT product development pipeline update process. The research team surveyed 18 product developer organizations, with an 83% response rate (*n* = 15). A desk review was then conducted, reviewing MPTs in all stages of development—both those already in the MPT Database and those identified through a supplementary literature review to ensure all new or emerging MPT candidates were reflected. A series of 28 in-depth semi-structured key informant interviews (KIIs) were then conducted with technical experts representing a vast array of HIV and STI prevention and contraception expertise. They included product developers, regulatory experts, program implementers, civil society leaders, policy makers, and donors/supporting agencies, among others. Respondents brought L/MIC perspectives from sub-Saharan Africa, Latin America, and the Asia Pacific Region. A pre-KII self-administered form and a KII interview guide were used to explore key informant input on missing/outdated research in the product developer surveys and desk review and other additional details on new or ongoing MPT research and development (R&D), as well as insights around priority MPT approaches and indications, key gaps and challenges, and recommendations for the field. Following the interviews, the research team aggregated and reviewed the interview notes to identify key themes (6).

## 3. Results

From the process described above, we propose six primary action strategies to advance MPTs for L/MICs, including identification of key research gaps and priorities.

### 3.1. Action area 1: technical challenges and opportunities

#### 3.1.1. Overview

The two basic design strategies for MPTs are: (1) formulation of a single drug capable of addressing two distinct indications, and (2) separate, independent drugs co-formulated into a single formulation. Details on different Active Pharmaceutical Ingredients (API) and delivery forms are summarized elsewhere (6–8).

Although antiviral drugs are the most common APIs used specifically for HIV prevention in MPTs, other single drug options are being evaluated for dual indications (9). Whereas established antiretroviral (ARV) drugs offer no protection against unintended pregnancy, some non-ARV drug products in development have dual indications (e.g., contraception + STIs) (7, 10). To date, the majority of the MPTs in development with a contraceptive indication are hormonal contraceptives, with expanding interest in non-hormonal contraceptive approaches (11). Aside from achieving an effective multiple API delivery system, it is equally critical that the MPT product configuration is also consistent with end-user preferences. Data from end-user studies have shown end-user dislike of daily dosing, and preference for longer-acting products (e.g., injectables, implants) (12), although on-demand products are of interests to some end-users too (13, 14). Importantly, evidence suggests that long-acting products are best aligned with end-user interest and adherence behaviors, which translate to better protection, consistent with findings from contraception research (15). However, ensuring the full suite of options is available to end users to enable choice is critical to meet the needs of diverse target populations with varied lifestyles and preferences. Other less familiar delivery types, including intravaginal rings (IVRs), gels, films, and non-daily oral tablets will likely expand the set of choices even further.

#### 3.1.2. Progress to date

##### 3.1.2.1. Status of MPT product pipeline

**Figure 1:**
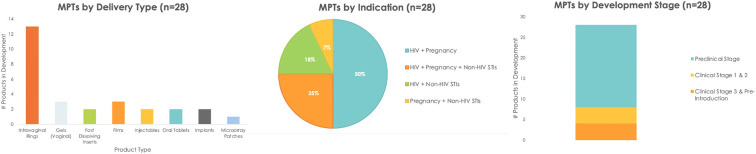
MPT product candidates by delivery type, indication and stage of development (as of December 2022) (6, 7).

The MPT pipeline is dynamic, with over two dozen MPTs encompassing eight delivery types, as shown in Figure 1 (7, 15–17). As of December 2022, more than half of the product candidates combined HIV prevention with contraception, and a third provided prevention against HIV and other STIs but without contraception.

Despite these advances, innovations in product design have remained conventional, resulting in redundancy of delivery forms. Half of the MPT pipeline is made up of IVRs, yet, many preference studies for potential MPTs highlight interest for provider-administered long-acting approaches, such as injectables and implants (18–20). Given the technical complexity of developing long-acting multi-drug formulations, few long-acting MPT approaches—aside from IVRs—are currently in the pipeline.

An important new trend is the expansion of APIs for MPTs. Since preventing HIV and unintended pregnancy was considered the initial MPT fieldwide priority, it evolved primarily from hormonal contraceptive and ARVs. This approach used drugs that were well-established (separately) for contraception and HIV treatment or prevention. Today, a number of alternative APIs are being evaluated in MPTs, including lectins, monoclonal antibodies (mAbs), and non-hormonal contraceptives (6).

##### 3.1.2.2. Status of end-user research

The preference for MPTs over single indication products is evident across diverse populations and geographies (19, 21). Lessons learned from the contraceptive field have shown that increasing available options to users improves overall method uptake and persistence, along with population-level coverage and beneficial health impact (22–24). Similarly, expert opinions, along with evidence from empirical studies, suggest that expanding PrEP and MPT options will improve prevention coverage and impact (25–27). Key preferences for end-users included ease of access, long(er)-acting delivery, discretion (i.e., use without partner knowledge), no impact on sex, and minimal side effects. Users also expressed preference for strategies that can easily gain partner approval, are de-medicalized, discreetly stored and transported, and packaged in visually appealing ways (19, 25–27). MPT acceptability research has mostly involved hypothetical preference and/or placebo studies. This acceptability gap is being filled with phase I trials assessing MPT rings (7), and more trials underway, including the oral dual prevention pill (DPP), after appropriate bioequivalence with individual doses of each drug is demonstrated (28).

##### 3.1.2.3. Recommended next steps

1. **Define criteria to evaluate MPT** product development efforts. This includes criteria to achieve an early “kill” on products with high risk and low probabilities of technical success or public health impact to optimize limited resources.
2. **Identify more potent APIs** to address all indications of relevance for MPTs and preferred delivery forms.
3. Expand **acceptability research with active MPT** products in the relevant female populations, (e.g., L/MICs at high risk, including AGYW).

### 3.2. Action area 2: addressing L/MIC regulatory pathways

#### 3.2.1. Overview

There is limited experience in multi-indication product development for MPTs. Regulatory standards for single indication products also apply to MPTs (e.g., safety, purity, stability, effectiveness, etc.). These standards will likely need to be achieved through experimental design that include combinations of APIs in the product (29).

In most L/MICs, navigating the local regulatory requirements governing registration of medicines including Chemistry, Manufacturing and Controls (CMC) is an ongoing challenge. Many product developers rely on the WHO Prequalification Program (PQ) to facilitate National Regulatory Authority (NRA) approvals in L/MICS (30). Additionally, Stringent Regulatory Authority (SRA) approvals can sometimes be leveraged, but LMICs may still need to complete their own regulatory reviews as well (31).

#### 3.2.2. Progress to date

In January 2022, the US FDA provided a guidance document for combination products (29). Several regulatory reviews, including their applicability to MPTs have highlighted areas to address (32–34). Single indication prevention products (e.g., dapivirine ring and oral PrEP) have gone through the regulatory process successfully and could serve as models to inform MPTs (35).

It remains unclear if the FDA Tentative Approval process, which was crucial for achieving affordable generic treatments for HIV, would apply to an MPT.

##### 3.2.2.1. Recommended next steps

1. MPT product development teams should be required by funders to include **appropriate regulatory expertise.**
2. With appropriate expertise in place, product developers should interact with **Stringent Regulatory Agencies (SRAs)** for input on product development strategies and plans.
3. MPT developers should request meetings and consultations with **in-country drug authority regulators** in L/MICs, to strengthen their capacity of independent review of dossiers, through regional meetings and consultations.

### 3.3. Action area 3: advancing MPTs from preclinical to clinical development

#### 3.3.1. Overview

MPT progression from preclinical to clinical development is similar to a single indication product. Data from studies relevant to CMC, safety studies in animal models, dose determination, pharmacokinetics (PK), and pharmacodynamics (PD) will be required. Additionally, determination of drug-drug interaction with an MPT will be needed. The FDA provides guidance on combination products, which should also be referenced (29). These guidance documents are relevant for cost estimations, cost effectiveness calculations, and risk reduction strategies.

Although early-stage funding exists for MPTs, support for promising MPT candidates beyond essential phase I studies is unclear. Most MPT candidates are in pre-clinical stages or phase 1 trials, largely supported by the National Institutes of Health (NIH) and United States Agency for International Development (USAID), and by small biotechnology companies. The pharmaceutical industry traditionally avoids acquiring and funding products until after phase II clinical development stages have been successfully completed to help “de-risk” their investment (36).

Furthermore, conducting randomized control trials (RCTs) of MPTs adds methodological complexity. Current standards for contraceptive and HIV prevention trial design differ widely, notably with the contraceptive Pearl Index approach (37) vs. the placebo or comparator product approach used for HIV. Needing to adequately power study arms to meet both may require large sample sizes.

#### 3.3.2. Progress to date

While the majority of MPTs in development are in pre-clinical development, several MPT candidates have progressed to phase I/ II, and two have progressed to phase 3 trials (Figure 1 and Table 1). R&D for long-acting MPT candidates was initiated, consistent with end-user preference data.

**Table 1:** Snapshot of MPTs in the R&D pipeline (*n* = 28).

Delivery type	# products in the pipeline	Delivery route		Administration		Advantages	Risks	Other considerations
		Systemic	Topical	Self	Provider			
Implants	2	✓			✓	Removable implants are technically simpler to develop. Yet, biodegradability may reduce healthcare provider (HCP) burden and improve user acceptability (e.g., less pain, scarring, no clinic visit required when product is spent)	Higher level of HCP training required; small surgical intervention to remove; lack of invisibility once placed	Leverage contraceptive trocars for insertion vs. developing unique insertion devices. Explore further users’ preference for removability vs. biodegradation
Rings	12		✓	✓		Can be self or provider inserted; immediately reversible upon removal; high acceptability among experienced users; rings exist for other indications (e.g., contraception, menopausal symptoms)	Unfamiliarity in target populations; partner detectability during sex; low prospective acceptability must be overcome with thorough education and training for first time users	May need HCP to administer first and/or verify placement; potential for OTC delivery
Long-acting injectables	2	✓			✓	High user compliance; discreet method; familiar delivery system	PK tail; silent infections; return to fertility concerns; pain at injection site	Requires access to qualified HCP for administration
Microarray patches	1	✓		TBD	TBD	Discreet method	*De novo* product; training for proper application; unfamiliarity in target populations; wait time at clinic prior to removal may be a concern	May need HCP to administer first and/or verify placement, or direct observation if self-placed at clinic
Oral pills	2	✓		✓		Affordable; stable; manufacturing simplicity; familiar delivery system	Adherence; low forgiveness if skipping; home storage needed	Pill and bottle/packet may be linked to HIV stigma
Gels (vaginal and rectal)	2		✓	✓		On-demand; affordable; stable; manufacturing simplicity	Adherence; messy; noticeability during sex	Potential for OTC delivery; delivered with reusable or disposable applicator
Films (vaginal)	3		✓	✓		On-demand; current R&D for extended-release formulation (1 month) without need for removal	Adherence; training for proper application; may stick to finger; unfamiliarity	May need HCP to administer first; potential for OTC delivery
Fast dissolving inserts (vaginal and rectal)	4		✓	✓		On-demand; can be dual compartment, potential for PrEP or PEP	Adherence	Potential for OTC delivery

##### 3.3.2.1. Recommended Next Steps

1. Assess the **technical and regulatory/development risks** for MPT products in the target populations which is largely achievable via International Council for Harmonization (ICH) Guidance on Risk Assessment and Resolution Strategies (38).
2. **Ensure that technical development is aligned with pre-defined milestones** for cost-effectiveness and end-user preferences.
3. Apply data from milestone steps 1&2 (above) to **justify continuation or termination of investments** during development.
4. Encourage open **collaboration between active funders** to assure complementary and appropriate investment in “best” MPT candidates. This process can be informed by existing funder collaborations (39–44).
5. Collaborate with scientists, researchers and regulators on **novel clinical trial designs** that can affordably evaluate multiple indication products in drug-device combinations.

### 3.4. Action area 4: cost and market potential

#### 3.4.1. Overview

Assessing product-market fit is essential for evaluating market potential and attracting investment for a novel MPT. Beyond establishing clinical efficacy, designing an MPT with attributes that are *affordable* to the eventual payers, *feasible* to deliver in the intended context and *attractive* to end users will increase the likelihood of finding a suitable market potential for investors. Assessing affordability ideally takes into account budgetary constraints, training needs for providers and cadres of staff for delivery/administration of the product, and the product's effectiveness.

#### 3.4.2. Progress to date

As noted above, there is a significant and growing body of literature on end user preferences that suggest significant market potential for MPTs. Discrete choice experiments have found preferences in a selection of sub-Saharan African countries for monthly injections over pills and rings (26, 27), which aligns with women's contraceptive familiarity and preferences (45).

Opportunities and challenges in costing products have also been identified (6) including the challenges of forecasting cost-of-goods (COGS) for large-scale manufacturing from pilot-scale prototypes, which has often then limited the ability to gain market traction. The importance of evaluating both cost and benefit from the perspective of the payers has also been identified (6). Health economics modelling on MPTs suggests that they will have the potential to be impactful and cost-effective, but such models are limited without real-world products (46–48). Procurement data from insurers and donors on family planning, HIV and STI products as well as end user willingness to pay studies can serve as an important benchmark for cost structure and potential pricing in different markets.

##### 3.4.2.1. Recommended next steps

1. Ensure that **target product profile criteria and standards** for MPTs are informed by evidence on willingness to purchase, ease of administration in L/MICs and end user preferences.
2. Expand and integrate **socio-behavioral & market research** into MPT R&D and introduction strategies, including from L/MICs.
3. **Optimize industry involvement** in MPT R&D to achieve scalability of products.
4. **Develop a path for MPT investment and introduction** that is relevant to public sector funders, private sector investors, and a range of markets.

### 3.5. Action area 5: market access

#### 3.5.1. Overview

Without early consideration and intervention, emerging MPTs will face challenges that impede timely uptake in L/MICs where many of the primary target populations live. Given the variety and complexity of MPTs, such as long-acting mechanisms and hybrid products, there will be additional market barriers to ensure equitable access. Affordability, supply capacity, intellectual property, regulatory pathways, adaptability, and usability are all key elements to be addressed in a timely manner to ensure delivery at scale.

#### 3.5.2. Progress to date

Initial efforts have established the investment case for MPTs (49) and MPT Target Population Identification Mapping Tool (50). These high-level advocacy tools are increasingly being bolstered by efforts to understand potential health and financial impact of specific technologies, such as the cost-effectiveness model for the DPP that is adaptable to other technologies (51).

To support early market access where prices are likely to be higher than desired, companies can pursue potential funders of market shaping financial mechanisms that can support manufacturing scale-up and faster price reductions, such as the Implant Volume Guarantee (52). Mechanisms developed in other health areas to support early identification of development and commercialization partners, as well as to enable voluntary licensing for generic manufacturing through entities like the Medicines Patent Pool (MPP), can also be leveraged (48).

##### 3.5.2.1. Recommended next steps

1. **Establish objective scientific and target product profile (TPP)-driven criteria and standards** to serve as benchmarks for MPT candidates that can foster supporting agency collaboration (52).
2. **Leverage co-sourced funding** within the public and private sectors to advance promising MPT candidates through the product development pipeline and to support manufacturing scale-up.

### 3.6. Action area 6: product introduction and rollout

#### 3.6.1. Overview

As no MPT has been launched in L/MICs since the male and female condoms, achieving successful market launch and scale will rely on close collaboration with a wide range of stakeholders to demonstrate the added benefits of a multi-indication product and to determine how best to integrate the product into the platforms available in family planning, sexual health and/or HIV. At a country level, national market authorization, inclusion in national treatment policies, and funding to implement rollout through national programs are essential to drive demand and support introduction (6). Transparent and affordable pricing is therefore crucial, as evidenced by the challenges currently faced with gaining local market authorizations and scale-up plans for long-acting injectable cabotegravir given its current higher cost than existing PrEP options (53).

Another potential challenge with MPTs is establishing the appropriate service delivery strategy. Currently, PrEP products are primarily serviced with end-users in primary health clinics and contraceptive products are managed via family planning clinics. Many countries in SSA are gradually integrating HIV testing and prevention into family planning clinics for efficient HIV testing, delivery of ARVs and contraception.

#### 3.6.2. Progress to date

The strategy for the launch of the DPP provides a roadmap to launch an MPT, inspired by similar efforts for other HIV and contraceptive products (48, 52, 54–57). However, several initial challenges that will affect the roll-out of MPTs exist, such as decision on who funds procurement and which supply chain is used. The USAID funded MOSAIC consortium, tasked with preparing for successful introduction of diverse PrEP options, could be leveraged (58).

Creating the enabling policy environment for MPTs is critical for success, and will benefit from growing efforts since the ECHO trial, to provide policy fora for the integration of HIV and family planning service delivery (59) Integrated SRH visits, using multi-service clinic facilities, and delivering products where a target market congregates are among the key approaches (6).

An MPT developer can benefit from strong architecture for product launches in both the family planning and the HIV space. Entities like the WHO, the Reproductive Health Supplies Commission, SEMA Reproductive Health, MOSAIC, and the ARV Procurement Working Group play important roles including guidelines development, market coordination and procurement forecasting. A wealth of in-country partners and platforms are available to support governments with training, demand generation and service delivery, such as MSI Reproductive Choices, DKT, the Global Fund and PEPFAR implementers.

##### 3.6.2.1. Recommended next steps

1. **Support implementation research and demonstration studies** which could provide important evidence to inform market authorization and offer insight into practicalities for end-users.
2. **Plan early for introduction and future adoption**. MPT awareness raising, promotion and training for end-users and health care providers is needed early to help ensure end-users and key stakeholders start thinking about MPTs well before they reach the market.
3. **Simplify access and method delivery**, including through the **self-care approach** (when possible). Offering a one-stop shop for multiple prevention needs, some user-controlled MPTs (depending on the delivery system and APIs) have the potential to expand self-care options for end-users, at least in the long term.
4. **Strategically select sites for Phase II and III MPT trials** where MPTs can be introduced and rolled out should they gain approval.
5. The **IND holder/sponsor of the product should lead the development and “own” the access plan** and incorporate access to intellectual property (IP) as part of development pathway.

## 4. Summary

This review provides a summary of the previously published 60+ page landscape of MPT product candidates in all stages of preclinical and clinical development (6). This review identifies six primary action strategies to advance MPT access in L/MICs and their progress to date. We also highlight key research gaps and priorities that can be addressed to strategically help advance the field.

Table 1 provides a snapshot of key considerations for the different MPTs currently in the pipeline. As the MPT field evolves, delivery types are expected to change. To realize the life-saving potential of MPTs, a strategic, collaborative and well-funded response to the gaps and next steps outlined in this paper is critical.
